# A targeted amplicon next-generation sequencing assay for tryptase genotyping to support personalized therapy in mast cell-related disorders

**DOI:** 10.1371/journal.pone.0291947

**Published:** 2024-02-09

**Authors:** Olga Li, Jason A. Hackney, David F. Choy, Diana Chang, Rhea Nersesian, Tracy L. Staton, Fang Cai, Shadi Toghi Eshghi

**Affiliations:** Genentech Research and Early Development, Genentech, Inc, South San Francisco, CA, United States of America; CNR, ITALY

## Abstract

Tryptase, the most abundant mast cell granule protein, is elevated in severe asthma patients independent of type 2 inflammation status. Higher active β tryptase allele counts are associated with higher levels of peripheral tryptase and lower clinical benefit from anti-IgE therapies. Tryptase is a therapeutic target of interest in severe asthma and chronic spontaneous urticaria. Active and inactive allele counts may enable stratification to assess response to therapies in asthmatic patient subpopulations. Tryptase gene loci *TPSAB1* and *TPSB2* have high levels of sequence identity, which makes genotyping a challenging task. Here, we report a targeted next-generation sequencing (NGS) assay and downstream bioinformatics analysis for determining polymorphisms at tryptase *TPSAB1* and *TPSB2* loci. Machine learning modeling using multiple polymorphisms in the tryptase loci was used to improve the accuracy of genotyping calls. The assay was tested and qualified on DNA extracted from whole blood of healthy donors and asthma patients, achieving accuracy of 96%, 96% and 94% for estimation of inactive α and βΙΙΙ^FS^ tryptase alleles and α duplication on *TPSAB1*, respectively. The reported NGS assay is a cost-effective method that is more efficient than Sanger sequencing and provides coverage to evaluate known as well as unreported tryptase polymorphisms.

## Introduction

Tryptase is the predominant secretory protease stored in granules and released by mast cells upon stimulation [1, 2]. Mast cell tryptase levels in bronchoalveolar lavage (BAL) fluid are elevated in asthma patients compared to healthy donors and correlate with disease severity [3]. Tryptase is elevated in the airways of severe asthma patients independent of type 2 biomarkers [3]. Tryptase genetics variations result in variability in the number of active tryptase β alleles, which in turn correlates with levels of tryptase in blood and BAL fluid [3]. *Ex vivo* assessment of primary mast cells from foreskins of human donors shows a correlation between tryptase activity per cell and number of active tryptase alleles [3]. Finally, asthma patients with higher tryptase β allele count may draw less benefit from anti-IgE therapy [3, 4]. These observations suggest tryptase as a potential therapeutic target for asthmatic patients who do not respond to available therapies. Also, they indicate the potential utility of tryptase genetics for identifying patients more likely to respond to different therapies. Enabling accurate genotyping of the tryptase genes in clinical studies is therefore essential to define the role of tryptase genetics and enable personalized treatments for therapies targeting mast cells in asthma.

**Fig 1:**
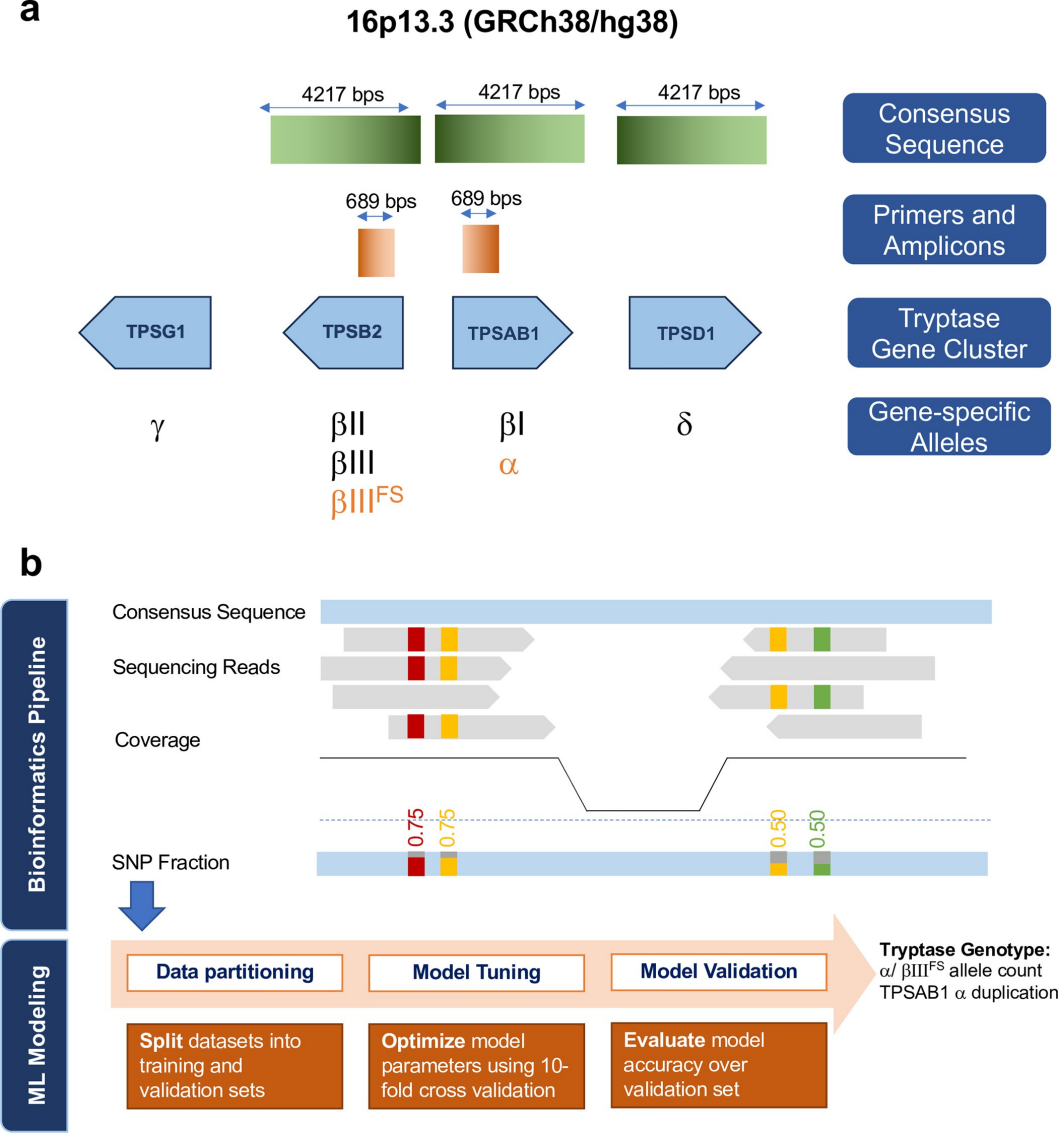
Schematic of tryptase gene cluster, PCR NGS analysis and tryptase genotyping workflow. a) A schematic of the Tryptase locus is shown. Tryptase locus on chromosome 16 comprises a highly homologous cluster of genes: *TPSAB1*, *TPSB2*, *TPSD1* and *TPSG1*. TPSAB1 and TPSD1 are located on the positive strand of DNA, while *TPSB2* and TPSG1 are on the negative strand. A consensus sequence that was generated for alignment of tryptase short reads in the PCR NGS workflow matches *TPSAB1*, *TPSB2* and *TPSD1* with high identity scores. Primers were designed to capture *TPSAB1* and *TPSB2*, and exclude *TPSD1* and *TPSG1*, resulting in two amplicons of 689 bps within *TPSAB1* and *TPSB2*. Various tryptase alleles expressed by different tryptase gene loci are shown. Inactive forms are highlighted in orange. b) The reads are first mapped to the tryptase consensus sequence. Fractional abundance of SNPs with sufficient sequencing coverage is calculated and reported as input features for developing the tryptase genotyping model. Highly correlated SNPs are removed from the feature set to reduce the number of redundant features. Data is split into training and testing sets and cross-validation and hyperparameter tuning are performed to optimize the model performance, while reducing model complexity and the risk of overfitting. The model is externally validated on the hold-out set. The colored SNPs represent the polymorphisms in tryptase that distinguish various alleles. bp: base pair. ML: machine learning.

Tryptase loci comprises a highly homologous cluster of genes on chromosome 16: *TPSAB1*, *TPSB2*, *TPSD1* and *TPSG1* (Fig 1A). In humans, enzymatically active soluble tryptase is encoded by *TPSAB1* and *TPSB2*. These two genes produce both active and inactive isoforms of tryptase. *TPSAB1* produces two isoforms, α and βΙ tryptase, while *TPSB2* produces βΙΙ, βΙΙΙ and βΙΙΙ^FS^ isoforms. α tryptase is an inactive isoform, resulting from amino acid changing mutations, which are thought to hinder autocatalytic activation and interfere with substrate binding [5, 6]. βΙ, βΙΙ and βΙΙΙ are active forms of tryptase [2, 5, 7–9]. A reported frameshift mutation in βΙΙΙ results in early truncation and produces another inactive isoform, βΙΙΙ^FS^ [10]. Additionally, duplication or triplication of α tryptase on *TPSAB1* has been reported, with variable clinical manifestations [11]. Tryptase genotyping aims to estimate the number of active β and inactive α and βΙΙΙFS alleles on the *TPSAB1* and *TPSB2* loci and detect the presence of α duplication on *TPSAB1*. Current evidence suggests that *TPSD1* and *TPSG1* do not produce soluble and enzymatically active tryptase, and are therefore excluded from genotyping.

Tryptase and other genes in regions of high homology pose major challenges for short-read sequencing technologies [12]. Inaccurate mapping of short read sequences to homologous regions of the reference genome leads to high genotyping errors. In recognition of this difficulty, the American College of Medical Genetics and Genomics’ guidelines requires laboratories to “develop strategies for detecting disease-causing variants within regions with known homology” [12, 13]. The standard Genome Analysis Toolkit (GATK) workflow is unable to accurately call variants in the tryptase region due to the aforementioned challenges. Also, no known commercial assays are available to allow for straightforward genotyping of the tryptase locus. A variety of PCR-based solutions may be used for genotyping. Commercially available or custom designed PCR-based Taqman assays are low-cost and efficient in detecting single-nucleotide polymorphisms (SNP) in a single PCR reaction [14]. Additionally, SNP microarrays can be used for high-throughput evaluation of thousands of SNPs and copy number variations (CNVs) [15, 16]. Despite their strengths and wide utility, some limitations have hindered the use of these assays for tryptase genotyping. For example, SNP microarrays may perform suboptimally in genetically diverse populations and Taqman assays have limited sequence coverage and are restricted to reported variants only. Moreover, the complexity of assay development due to the high level of sequence identity between tryptase genes might be an impeding factor. Potential duplication or triplication of α tryptase on *TPSAB1* further complicates assay development. We previously employed a PCR-based Sanger sequencing approach to sequence distinctive partial α and β tryptase regions and deduce the number of active β tryptase alleles. This assay, although accurate, is low throughput and unable to detect *TPSAB1* copy number variation [3, 17], necessitating a more high-throughput and robust assay for implementation in clinical studies. Additionally, we developed a whole genome sequencing (WGS)-based assay, which was able to determine active β allele count as well as copy number variation in *TPSAB1* locus. The assay however is costly, thus limiting its utility for clinical sample testing.

Here, we report a targeted amplicon NGS assay and downstream bioinformatics analysis to estimate the number of active β and inactive α and βΙΙΙ^FS^ alleles and α duplication in DNA extracts from whole blood. This assay is a more robust and cost-efficient alternative to Sanger sequencing that relies on targeted amplification and sequencing of the tryptase loci, mapping the sequencing reads to a consensus *TPSAB1* and *TPSB2* reference sequence and estimating the fractional abundance of common polymorphisms in the amplicon region. Machine learning classification uses the polymorphism fractional abundances as input to build a multivariate model for estimating the number of α and βΙΙΙ^FS^ alleles as well as presence of α duplication in the genomic DNA (Fig 1B). Number of active β alleles are inferred from total and inactive tryptase alleles. Here we report the process for developing and qualifying the assay as well as the analytics pipeline. Through targeted PCR amplification, this assay ensures that sufficient sequencing depth is achieved across the length of the tryptase gene. Additionally, this assay not only enables genotyping of the tryptase loci based on reported polymorphisms, but also allows for future evaluation of unreported functional polymorphisms that may not be captured with methods that require pre-specification of polymorphisms such as droplet digital PCR. High fidelity quantitation of active β tryptase alleles may enable real-time genotyping of patients for targeted therapies in mast cell-driven diseases.

## Materials and method

### Whole blood collection and DNA preparation

Whole blood samples were collected from 18 healthy donors and 73 asthma patients. Samples were selected to increase diversity of tryptase genotype. Table 1 shows an overview of subject participants and datasets in this study. Briefly, 6 ml whole blood samples were collected in BD Vacutainer tubes (BD 68661) per manufacturer’s instructions. Samples were stored at -20°C prior to DNA extraction. DNA was extracted from samples collected prior to 2013 using the Chemagic DNA kit (Perkin Elmer, Waltham, MA). Samples collected after 2013 were extracted using the MagNA Pure 96 (Roche Life Science, Penzberg, Germany). Samples were processed in four batches.

**Table 1:** Summary of datasets for development and validation of the tryptase PCR NGS assay pipeline. Samples from healthy donors and asthma patients were included. Technical replicates were run on samples from DS2. Samples were selected based on tryptase genotype and population diversity. DS3 includes subjects from a diverse population of African, American, East Asian, European and South Asian ancestry.

Dataset	Size	Samples	Ancestry	Application
**DS1**	18	Healthy Donors	Not Available	Validation
**DS2 (rep1)**	38	Asthma	European	Training
**DS2 (rep2)**	38	Asthma	European	Validation
**DS3**	35	Healthy Donors and Asthma	Diverse	Validation

### PCR NGS libraries and sequencing

A schematic of the targeted amplicon sequencing workflow is summarized in S1a Table in S1 File. The tryptase amplicon that captures *TPSAB1* and *TPSB2* and excludes *TBSD1* and *TPSG1* is depicted in Fig 1A and S1 Fig in S1 File.

An Illumina targeted sequencing strategy was employed to amplify the tryptase region of interest using the target specific primers (S1b Table in S1 File) with overhang adapters (S1c Table in S1 File). The first stage PCR was performed using 1.5 μl of 10 μM forward primer, 1.5 μl of 10 μM reverse primer, 100 ng of template genomic DNA, and 25 μl of KAPA HiFi HotStart ReadyMix per 50 μl reaction using the cycling settings listed in S1d Table in S1 File. Amplified products were visualized using TapeStation and confirmed by size determination. Subsequently, the samples were purified using AMPure XP beads at 0.7x ratio for a 50 μl elution volume and concentration was determined by Qubit dsDNA BR kit. Library amplification was carried out by combining 25 μl of purified PCR product with 10 μl pre-paired i7 and i5 index adapters with 15 μl of Nextera PCR Master Mix per 50 μl reaction using the cycling settings listed in S1e Table in S1 File. The purified libraries were visualized by TapeStation again. Next, libraries were quantified by qPCR using the KAPA SYBR FAST Universal qPCR Kit with Illumina Primer Premix on Biomek FX. Libraries were then normalized to 2 nM, pooled, and sequenced by Illumina MiSeq targeting two 300 bp regions using paired-end sequencing. PhiX control was added to the library to increase diversity of the highly homogenous tryptase DNA amplicon and to serve as a QC control as recommended by Illumina. The final sequencing data comprises a total of 689 bp after clipping low quality bases. The sequence includes two 300 bp amplicons, with a <100 bp gap of coverage.

### Sanger sequencing

Sanger sequencing was performed on a subset of samples as previously described to serve as a gold standard for developing and qualifying the PCR NGS assay. Briefly, genomic DNA was amplified by PCR followed by Sanger sequencing. The intensity ratio of two (A/B) alleles was used to determine the genotype. The assigned genotypes were visually confirmed for 5% of the population without error [3, 10, 18].

### Whole genome sequencing (WGS)

WGS data collected from a subset of the samples and a fit-for-purpose pipeline as described before [3] was used as a second confirmatory method of calling tryptase genotypes. Briefly, WGS data was aligned to the human reference genome (GRCh38) using Burrows-Wheeler Aligner (BWA) and reads aligning to the tryptase loci (chr16:1200000–1300000) were extracted and realigned to the tryptase consensus sequence. The proportion of reads consistent with the frameshift in the *TPSB2* gene was used to estimate the number of βΙΙΙ^FS^ tryptase alleles. A linear combination of α tryptase-specific SNPs were used to estimate the number of α alleles and α duplication in the *TPSAB1* gene. Tryptase genotypes determined by Sanger and WGS sequencing were in strong agreement [3] and were used for training and evaluation of the PCR NGS pipeline.

### ddPCR analysis

A custom semi-quantitative ddPCR assay was used for tryptase genotyping. Genomic DNA was extracted from whole blood using the QIAamp DNA Blood Midi Kit. Extracted DNA was quantified by Qubit and normalized to 5ng/uL. Extracted DNA (25 ng/reaction) was used as a template in three ddPCR reactions directed towards tryptase α, tryptase βΙ/ΙΙ/ΙΙΙ and tryptase βΙΙΙ^FS^. gDNA was digested during the reaction set up with BamH1 (digestion for tryptase α and βΙ/ΙΙ/ΙΙΙ) or BgIII (digestion for tryptase βΙΙΙ^FS^), which cut at the 3’ end of *TPSB2* to ensure that the *TPSB2* and *TPSAB1* loci were separated into separate droplets. The primers and probes for tryptase α and βΙ/ΙΙ/ΙΙΙ were taken from Lyons, et al [17]. The forward and reverse primers were the same for both reactions, whereas detection specificity was conferred by the probes.

The tryptase βΙ/ΙΙ/ΙΙΙ assay does not distinguish between βΙΙΙ and βΙΙΙ^FS^. Therefore, a custom set of primer-probes were used in a separate ddPCR reaction for βΙΙΙ^FS^. Primers and probes for the AP3B1 gene were included in each reaction to serve as control for copy number normalization (dHsaCP1000001 assay by Bio-Rad). Following reaction set up, the ddPCR reaction was partitioned into nanoliter sized water in oil droplets using the QX200 Droplet Generator and subsequently amplified by PCR. Amplified samples were incubated at 4°C for at least 30 mins before being quantified on the QX200 Droplet Reader, which flew the droplets in single file past a two-color optical detection system to determine which droplets were positive for the target being assayed. Using the QuantaSoft Software, Poisson statistics were applied to the data to determine the number of alleles of each tryptase isoform, which was determined by concentration of tryptase specific copies divided by concentration of AP3B1 copies multiplied by 2. Copy numbers were rounded to the nearest tenth, and the number of alleles for each tryptase isoform was reported as 0, 1,2,3, or > = 4. Each run contained a positive control sample with known tryptase isoform alleles for each reaction, as well as a no-template control. Wells with fewer than 10000 total events were considered inadequate and repeated. If the copy number for any tryptase reactions fell between the established range for an allele determination, the sample was repeated.

### PCR NGS data analysis

A schematic workflow of the PCR NGS tryptase genotyping workflow is depicted in Fig 1B. Sequencing reads from amplicon sequencing were realigned to a consensus sequence extracted from overlaying *TPSAB1*, *TPSB2* and *TPSD1* gene loci. Realignment was done using BWA and in accordance with best practices from GATK. The consensus sequence (S1 Fig in S1 File) covered exon and intron regions of *TPSAB1*/*TPSB2* and included the loss-of-function mutations in the α allele and the frameshift observed in βΙΙΙ^FS^ tryptase allele. The sequencing depth at each base pair was estimated and used to remove SNPs with low sequencing depth from further analysis. The fractional abundance of each SNP was estimated after realignment to the consensus sequence. The estimated fractions were used as input features to supervised machine learning models for estimation of inactive α allele count, inactive βΙΙΙ^FS^ allele count and duplication of α allele on the *TPSAB1* loci (Fig 1B).

### Tryptase genotyping model training and validation

A multi-class logistic regression model [19] was trained to estimate the number of α tryptase alleles. A binary logistic regression model was developed to identify samples with germline α duplication in the *TPSAB1* gene. Genotypes generated from Sanger or WGS sequencing of matching samples were used as ground truth and for training the model. Data was randomly split into training and testing sets. Repeated 5-fold cross validation (n = 3) on the training set was used for model tuning. The model was externally validated on three independently analyzed batches of samples as outlined in Table 1. βΙΙΙ^FS^ allele count was simply determined by presence or absence of the frameshift single-nucleotide variant (SNV). The glmnet package was used for fitting the regularized logistic regression model [20] and model training and validation was done using the Caret package [21] in R. All analyses were performed in R (Fig 1B).

### In silico assessment of the impact of sequencing depth on genotyping accuracy

For each sample, reads mapped to the tryptase region were randomly subsampled to create simulated *in silico* samples with varying levels of sequencing depth to simulate the effect of reduced depth. The process was repeated 10 times for each sample at each simulated depth and the SNP fractional abundances were recalculated for each of the in silico samples. The tryptase genotyping models were applied to each simulated sample and model accuracies were estimated at each simulated depth for training and validation sets. Mean and standard deviation of genotyping accuracy were calculated for each model and dataset.

### Ethics approval and consent to participate

Moderate asthmatic patients were participants of several clinical studies of Lebrikizumab (LUTE; clinical-trials.gov: NCT01545440, VERSE; clinical-trials.gov: NCT01545453, MILLY; clinical-trials.gov: NCT00930163, LOVALTA I; clinicaltrialsregister.eu: GB28688, LOVALTA II; clinicaltrialsregister.eu: GB28689). Additional healthy human control blood and DNA samples were obtained through an employee donation program at Genentech Health Services (South San Francisco, CA, USA). Approval was obtained from the Western Institutional Review Board (WIRB Protocol #20080040). All clinical study participants included in these analyses reviewed and consented to research by signing Informed Consent Forms, which were reviewed and approved by local Institutional Review Board or Ethics Committees. All methods were carried out in accordance with the Declaration of Helsinki Principles.

## Results

### Genotyping cohorts

Three sample sets analyzed in separate batches were used for development and validation of the PCR NGS assay and the analytics pipeline (Table 1). Samples were selected to maximize diversity of tryptase genotypes tested. Significant differences in tryptase genotypes and number of α, β and βΙΙΙ^FS^ alleles have been reported in different human populations [10]. To assess the performance of the genotyping assay in ethnically diverse populations, samples from subjects of African (AFR), American (AMR), East Asian (EAS), European (EUR) and South Asian (SAS) ancestry were included (DS3 in Table 1). Technical reproducibility of the assay was evaluated by processing a sample set in two technical replicates, starting from DNA extraction (DS2, Rep1 and Rep2). The models for estimating the inactive tryptase alleles and copy number variation in *TPSAB1* were developed on dataset DS2, first technical replicate (N = 38) and independently validated on the DS1, DS3 and DS2 (second technical replicate) datasets.

### Data quality assessment

For each dataset, sequencing depth was assessed by counting the number of *TPSAB1* and *TPSB2* reads aligned to the tryptase consensus sequence. The sequencing depth was used to assess the quality of individual samples and sequencing and remove low quality samples from further analysis as seen in S2 Fig in S1 File. Sequencing depth achieved from targeted NGS surpassed that of WGS at about one-tenth of the cost, increasing the confidence in identifying SNVs and quantifying their relative abundance. For example, we observed sequencing depths of 20,000 to 800,000 in the tryptase loci sequenced by PCR NGS across the datasets included in this study, compared to 0–400 achieved from WGS reads. However, the PCR NGS sequencing depth was not uniform across the length of the consensus sequence (S2 Fig in S1 File). The assay covers two amplicon regions of 300 bps each, resulting in a <100 bp gap of coverage in the middle of the consensus sequence.

### Tryptase genotyping model

We aimed to develop a model to estimate the number of α and βΙΙΙ^FS^ tryptase alleles from fractional abundance of SNPs in the *TPSAB1* and *TPSB2* loci. *TPSAB1* encodes the genes for inactive α and active βΙ tryptase, whereas *TPSB2* encodes the genes for active βΙΙ and βΙΙΙ and inactive βΙΙΙ^FS^ tryptase alleles. Therefore, depending on *TPSAB1* and *TPSB2* genotype, each patient may have 0, 1 or 2 α tryptase and 0, 1 or 2 βΙΙΙ^FS^ tryptase alleles. S2 Table in S1 File shows examples of the expected number of α, βΙΙΙ^FS^, and active β alleles for different genotypes. Multi-class logistic regression model was selected for estimation of α allele count in the absence of α CNV (S2 Table in S1 File). Since βΙΙΙ^FS^ is determined by a single frameshift in the *TPSB2* gene, a simple linear model was used to determine the number of βΙΙΙ^FS^ alleles. Presence of α duplication was determined by a binary logistic regression model.

Fractional abundance of tryptase SNPs mapped onto the consensus sequence were used as features for model development to determine number of α, βΙΙΙ^FS^ and presence of α tryptase duplication. Tryptase genotypes determined by Sanger or WGS sequencing were used as reference for supervised training. To ensure that only SNPs with high confidence coverage were included in the modeling, a minimum threshold of 100 reads was applied to remove SNPs with insufficient sequencing depth. The vast majority of the observed tryptase SNPs were co-inherited as haplotypes and therefore were highly correlated (S3 Fig in S1 File). Therefore, after prioritizing loss-of-function and allele differentiating SNPs, features that were highly correlated with the prioritized SNPs were further removed from the list of features, as they provide only minimal additional information at best. A correlation coefficient of 0.6 or higher was used to filter out the highly correlated features. These SNP prioritization steps helped significantly reduce the number of input features from more than 200 to 12. Fractional abundances of the remaining prioritized SNPs were included in the model as input features. Cross-validation was used for tuning the model hyperparameters and optimizing the performance. Regularization was used to penalize the model for adding SNPs with minimal improvement to the model performance, thus favoring simpler models with fewer features to complex and potentially overfitting models. For each prediction, the final model was selected based on highest accuracy. Regularization helped condense the most informative features incorporated into the model to only 3 SNPs. A summary of the models’ attributes and SNPs included in each model are provided in Table 2 and coefficients of the final models are shown in S4 Fig in S1 File.

**Table 2:** Summary of tryptase genotyping models, SNPs and model performance.

Model	SNPs	GRCh38 position	Model Accuracy Training and Validation Sets (%)	Model Accuracy Validation Sets (%)
α tryptase allele count	rs146223687 rs200858385 rs547415829	ch16: 1241174 ch16: 1241268 ch16: 1241533	96.1% (123/128)	95.6% (86/90)
βΙΙΙ^FS^ tryptase allele count	rs1217155915	ch16: 1291647	96.1% (123/128)	96.7% (87/90)
α tryptase duplication on *TPSAB1*	rs146223687 rs200858385	ch16: 1241174 ch16: 1241268	94.3% (99/105)	94.0% (63/67)

To validate the model, the performance was evaluated on three datasets including a technical replicate of the training set and two independent datasets as shown in Table 1. On average, the models estimated α count, βΙΙΙ^FS^ count and α duplication with accuracies of 96%, 96% and 94% across the four datasets in this study. The α duplication model achieved precision of 92% across the four datasets. Estimating the performance of the model on the validation set alone yielded similar prediction performance (Table 3). No notable difference was observed in genotyping accuracy between subjects from diverse populations in DS3. Comparing the workflow on two technical replicates show strong concordance of 97–100%, indicating high technical reproducibility (Table 4). Additionally, we observed no evidence of higher genotyping error in subjects of non-European ancestry, indicating the workflow can be used for tryptase genotyping in diverse populations. Finally, *in silico* simulations were performed to quantitatively assess the depth requirements for accurate genotyping. To do so, the effect of reduced sequencing depth was simulated through subsampling of aligned reads, creating in silico samples with varying sequencing depths. Assessing the relationship between depth and genotyping accuracy showed that the α tryptase allele and α duplication models performed equally well with depths as low as ~2000 and the βΙΙΙ^FS^ model performs equivalently at depth of ~1000 (S5 Fig in S1 File).

**Table 3:** Accuracy of the PCR NGS tryptase genotyping workflow. The genotyping workflow achieves an estimated accuracy of 95% across roughly 130 samples analyzed in 4 batches.

N = 128	α allele count (reference)				N = 128	βΙΙΙ^FS^ allele count (reference)				N = 105	α duplication (reference)		
α allele count (prediction)		**0**	**1**	**2**	βΙΙΙ^FS^ allele count (prediction)		**0**	**1**	**2**	α duplication (prediction)		**No**	**Yes**
	**0**	37	1	1		**0**	71	0	1		**No**	75	4
	**1**	1	52	1		**1**	1	40	0		**Yes**	2	24
	**2**	0	1	34		**2**	1	2	12				

**Table 4:** Technical reproducibility of the PCR NGS tryptase genotyping workflow. Genotypes reported by the workflow are highly concordant between two technical replicates of sample set DS2.

N = 37	α allele count (Technical Rep1)				N = 37	βΙΙΙ^FS^ allele count (Technical Rep1)				N = 37	α duplication (Technical Rep1)		
α allele count (Technical Rep2)		**0**	**1**	**2**	βΙΙΙ^FS^ allele count (Technical Rep2)		**0**	**1**	**2**	α duplication (Technical Rep2)		**No**	**Yes**
	**0**	11	0	0		**0**	20	0	0		**No**	26	0
	**1**	0	14	1		**1**	0	12	0		**Yes**	1	10
	**2**	0	0	11		**2**	0	0	5				

The model performance was additionally validated against tryptase genotyping using digital droplet PCR analysis on a subset of the samples. The ddPCR assay reported the total number of α copies, total number of βΙ/ΙΙ/ΙΙΙ/ΙΙΙ^FS^ and number of βΙΙΙ^FS^ copies. However, it did not explicitly report α duplication. To compare the PCR NGS genotyping model output with the ddPCR assay readout, total number of α and βΙ/ΙΙ/ΙΙΙ/ΙΙΙ^FS^ copies were derived from the output of the PCR NGS workflow (S3 Table in S1 File). The output of the ddPCR assay and the PCR NGS model were highly concordant resulting in 92% (47/51), 100% (51/51) and 94% (48/51) identity in total α copies, βΙΙΙ^FS^ copies and total number of βΙ/ΙΙ/ΙΙΙ/ΙΙΙ^FS^ copies over 51 datapoints, respectively (S4 Table in S1 File).

The fractional abundance of polymorphisms in the tryptase loci inform the relative number of alleles in this region. Prior to model development, feature selection centered on inclusion of loss-of-function tryptase mutations as essential predictors. These include a point mutation in the propeptide region of α tryptase (Arg to Gln) (rs146223687), which renders it catalytically inactive and a frameshift in βΙΙΙ tryptase (rs1217155915), which results in early truncation of the protein. After removing highly correlated polymorphisms to reduce informational redundancy, the training process supplements the core features with additional tryptase polymorphisms in an unbiased manner to optimize the model performance. Through this process rs200858385 and rs547415829 are selected by the algorithm in an unbiased manner as additional features of importance for estimation of α allele and α duplication (Table 2 and S4 Fig in S1 File). Both of these polymorphisms are synonymous mutations in the coding region of *TPSAB1*. In the absence of α duplication, the *TPSAB1* loss-of-function SNP (rs146223687) is sufficient to determine the number of α alleles. However, the presence of copy number variations in *TPSAB1* changes the distribution of this SNP and necessitates addition of other *TPSAB1* polymorphisms to resolve not only the number of α alleles, but also the presence of α duplication (S6 Fig in S1 File).

## Discussion

Knowledge of tryptase genotype may inform personalized treatment for tryptase-mediated diseases. Here, we report a PCR NGS workflow for accurate and precise genotyping of tryptase. PCR NGS output was 95% concordant with reference genotypes determined by WGS and/or Sanger sequencing and performed equally well in patient samples from ethnically diverse populations. The PCR NGS workflow has superior throughput compared to Sanger sequencing. Moreover, in contrast to Sanger sequencing, it enables identification of α copy number variation. Increased germline *TPSAB1* α copy numbers leads to α-tryptasemia and elevated serum tryptase and is associated with multisystem disorders, where severity of the symptoms correlates with the number of *TPSAB1* α copy number [3, 11]. On the other hand, PCR NGS can be performed at about one-tenth of the cost of WGS. The cost can be significantly reduced through sample multiplexing if permitted by the study design, for example in the case of retrospective sample processing. Therefore, PCR NGS is a more cost-efficient option, while offering the same level of information on tryptase genotypes. By targeting the tryptase loci, the PCR NGS approach allows for deeper sequencing and improved sensitivity for quantifying tryptase polymorphisms. In fact, upon deeper investigation of samples where WGS and PCR NGS reported different genotypes, we found the PCR NGS method to be more likely to be correct in the majority of discrepant cases (S6 Fig in S1 File). For example, samples with loss-of-function α SNP (rs146223687) fraction of 0 lack any α alleles, whereas the reference method reports non-zero α allele counts in at least two such samples (S6a Fig in S1 File). Additionally, the WGS pipeline is unable to detect α duplication in at least two samples, which according to their α SNP fractional abundance appear to have duplication in *TPSAB1* (S6b Fig in S1 File). Finally, the relative abundance of the frameshift modification on *TPSB2* should determine the number of βΙΙΙ^FS^ alleles, however, several samples appear to be mislabeled by the reference method (S6c Fig in S1 File), which could be attributed to the superior sensitivity of PCR NGS assay in quantifying the fractional abundance of polymorphisms across the tryptase loci. Additionally, S6a and S6b Fig in S1 File show that rs146223687, the most commonly used SNP for determining α tryptase, is not sufficient for accurate determination of α tryptase allele count and α duplication. This is further demonstrated by the improvement achieved through addition of rs200858385 and rs547415829.

Digital droplet PCR (ddPCR) is another sensitive and precise platform for quantifying tryptase allele copies and detecting α duplication in *TPSAB1*. ddPCR assays have been extensively used for pathogen detection, mutation monitoring and analysis of gene copy number variants [22–24]. Estimating α and βΙΙΙ^FS^ requires a multiplexed ddPCR assay targeting loss-of-function SNPs in the *TPSAB1* and *TPSB2* loci. Both the ddPCR and PCR NGS assays are robust and sensitive methods for tryptase genotyping. Comparison of results on a subset of ~50 datapoints shows high levels of agreement between the two methods. A few differences should be noted between these two methods that can inform selection of the most appropriate platform for each application. In the clinical trial setting, the ddPCR assay is more cost-effective, in particular for prospective testing, when there may be a need for real-time processing of the samples to determine patient eligibility or stratification. The ddPCR computational workflow is more straightforward and does not require bioinformatics expertise. On the other hand, ddPCR requires pre-specification of SNPs for assay design and validation and therefore can only quantify pre-specified alleles, whereas the PCR NGS platform allows for sequencing of the tryptase loci, determined by the primers, in an unbiased way. The main advantage of such an unbiased approach is the ability to query the data for non-specified alleles, which can be valuable for exploratory and reverse translational research. For example, the NGS data could be used to not only estimate active β alleles but also differentiate between βΙ and βΙΙ alleles.

Long-read sequencing (LRS) is an evolving technology that could potentially address some of the limitations of short-read sequencing. By covering longer reads, spanning between several kilobases to megabases of DNA, LRS can improve mapping of homologous sequences to the reference genome, thus improving the accuracy of genotyping in such regions [25], as for example demonstrated for SMN1/SMN2 and HSPA1A/HSPA1B genes [26]. For tryptase genotyping, the technology should in principle enable identification of α polymorphism and duplication in *TPSAB1* and the frameshift in the *TBSB2* regions. Despite their advantages, many of the current LRS technologies suffer from high error rates compared with short-read NGS, which has limited their application in diagnostic settings [27, 28]. Polishing tools have been shown to improve the error rate as have recent developments in base-calling algorithms and new data types, such as high-fidelity (HiFi) reads [25], making this technology a promising tool for tryptase genotyping, which will require extensive development and validation prior to use.

The advantages of the PCR NGS workflow mark this assay as a valuable tool to decipher tryptase genotypes from genomic DNA extracted from whole blood and to facilitate studying the role of tryptase genetics in mast cell-mediated disorders in clinical and research settings. Additionally, the proposed framework for mapping reads to a consensus sequence may be used in genotyping of other highly homologous gene loci.

## Supporting information

S1 File Supplementary figures and tables. This file contains the supplementary figures and tables referenced in this study.
(PDF)

S2 File Tryptase genotyping modeling data. This file contains the fractional abundances for the SNPs rs146223687, rs200858385, rs547415829 in Table 2, and predicted and reference α and βIII^FS^ alleles and α duplication. DS1, DS2 –Rep1, DS2 –Rep2 and DS3 are included.
(CSV)
