# Supplementary material for: A targeted amplicon next-generation sequencing assay for tryptase genotyping to support personalized therapy in mast cell-related disorders
Source: PLoS One. 2024 Feb 9;19(2):e0291947. doi: 10.1371/journal.pone.0291947 (PMC10857577; doi:10.1371/journal.pone.0291947)
Supplement: S1 File — This file contains the supplementary figures and tables referenced in this study. (PDF) [file pone.0291947.s001.pdf]

A targeted amplicon next-generation sequencing assay for Tryptase genotyping to support personalized therapy in mast cell-related disorders

Supporting Material

Olga Li[1&], Jason A. Hackney[1&], David F. Choy[1], Diana Chang[1], Rhea Nersesian[1], Tracy L. Staton[1], Fang Cai[1], Shadi Toghi Eshghi[1*]

Affiliations:

[1]Genentech Research and Early Development, Genentech, Inc, South San Francisco, CA, USA

[*]Corresponding author: toghiess@gene.com

[&]These authors contributed equally.

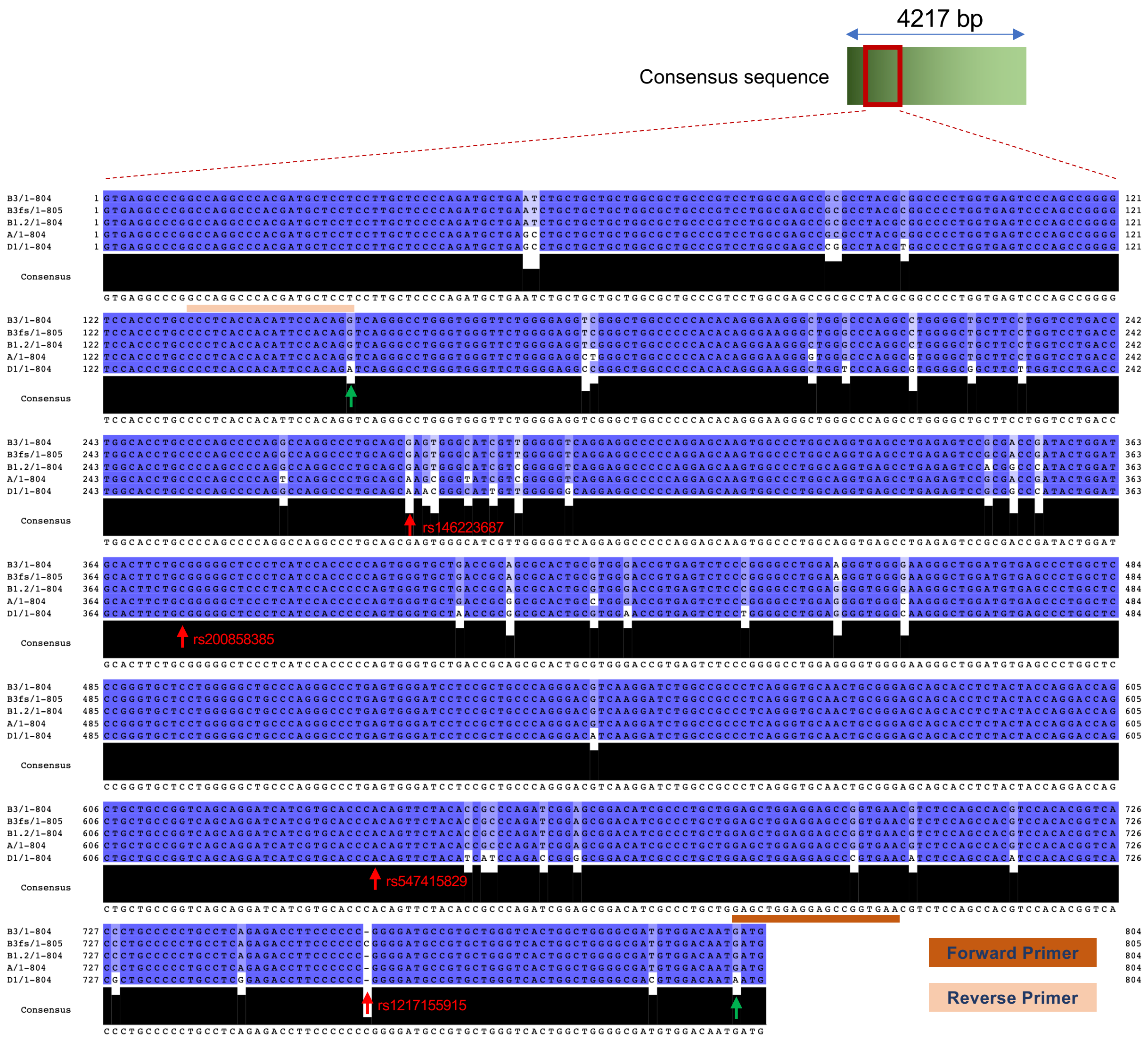

S1 Fig. Consensus sequence from alignment of *TPSAB1*, *TPSB2*, and *TPSD1* genes. A consensus sequence is built for alignment of tryptase reads. Part of the consensus sequence covering the targeted amplicon region is expanded. B3, B3fs, B1.2, A and D1 correspond to βIII, $βIII^{FS}$, βI/βII, α and δ alleles, respectively. Lighter shades highlight variations between the sequences. Consensus at each base is determined by majority voting between βIII, $βIII^{FS}$, βI/βII, α and δ alleles. Forward and reverse primers are displayed above the sequence and indicated by dark and light orange, respectively. Green arrows marks SNPs in the forward and reverse primers that allow for exclusion of *TPSD1*. Red arrows mark the SNPs that were used in the genotyping models listed in Table 2.

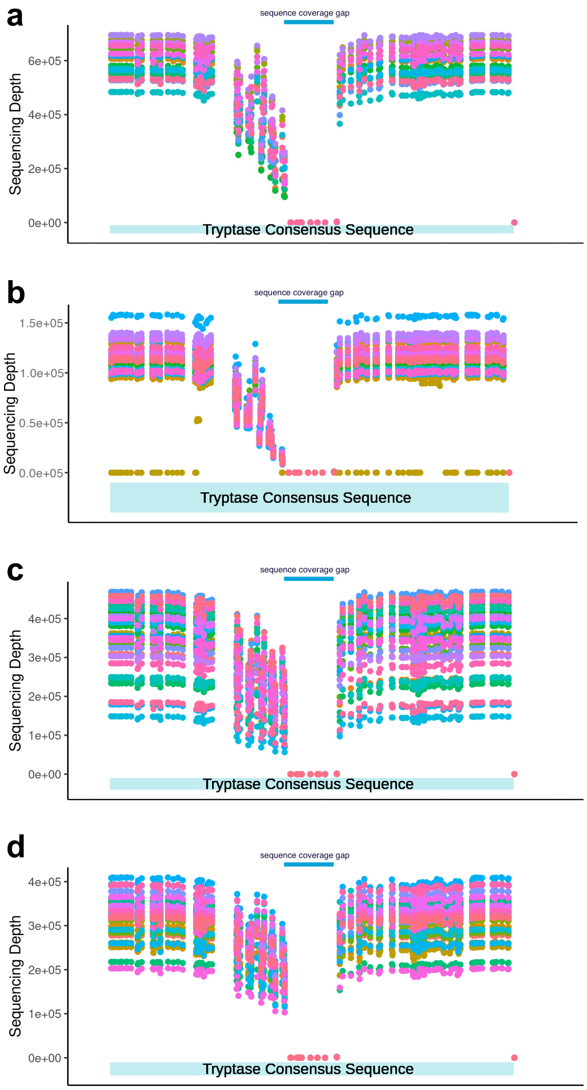

S2 Fig. Tryptase reads aligned to the consensus sequence. Cumulative number of reads corresponding to *TPSAB1* and *TPSB2* aligned to the tryptase consensus sequence for a) DS1, b) DS2, replicate 1, c) DS2, replicate 2 and d) DS3 are depicted. Each dot represents an SNV and each color represents a unique sample. The x axis represents the consensus sequence in the amplicon region. This figure can help identify low quality samples. Primers are designed to target a 689 bp region of the genome through two 300 bp amplicons, resulting in a gap in coverage between the two amplicons.

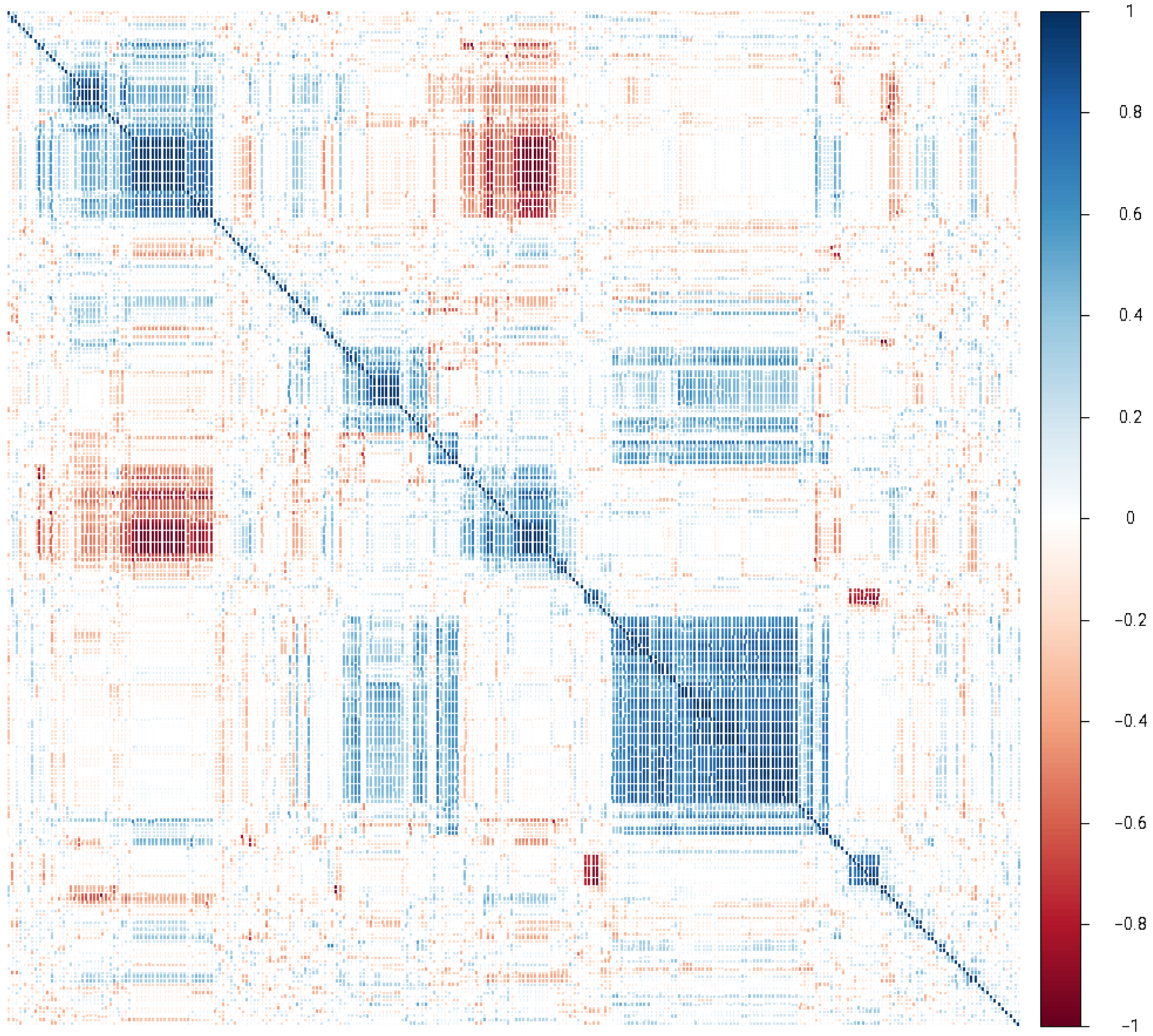

S3 Fig. Correlation plot of SNP fractional abundance on the tryptase loci. Representative example from DS2, rep 1 is depicted. Each row and column correspond to an SNV and the heatmap color indicates the correlation coefficient between fractional abundance for each pair of SNVs with blue indicating positive and red indicating negative correlation. As expected, correlation of clusters of SNPs on haplotypes result in high levels of information redundancy and therefore the SNPs are first reduced to a smaller set of non-correlating features before model development and tuning.

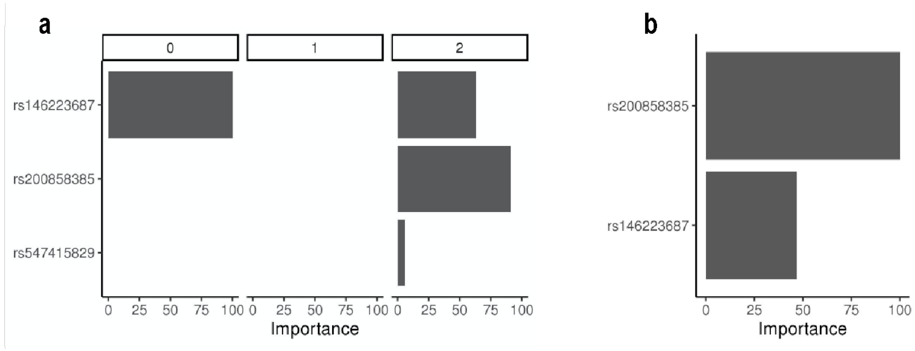

S4 Fig. Importance of individual SNPs in the tryptase genotyping for a) α allele count and b) α duplication. Bars indicates the absolute value of the coefficients for each SNP fraction in the final model. The coefficients were extracted from the model using the varImp function in the R caret package.

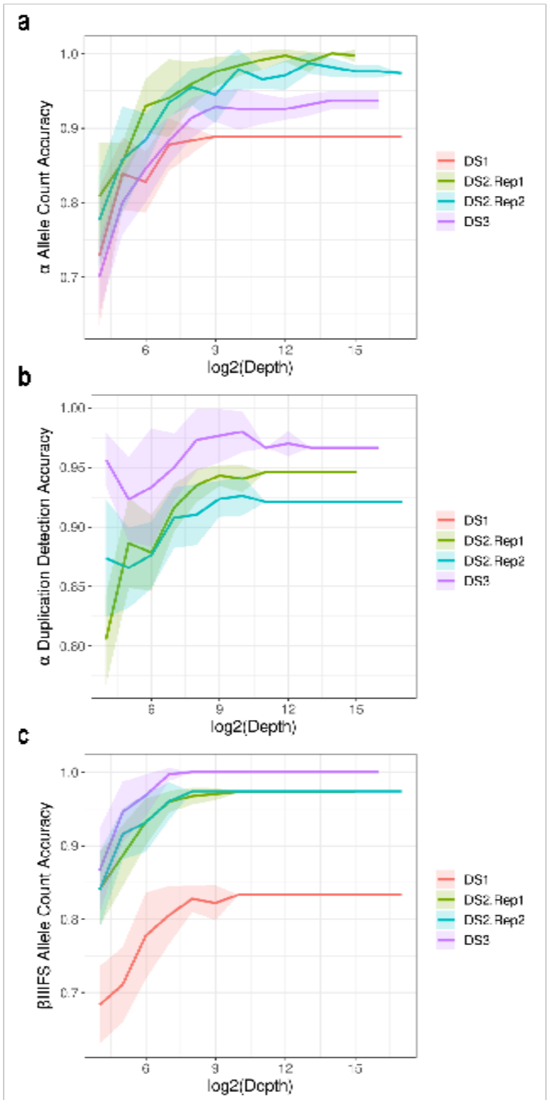

S5 Fig. Model accuracy vs sequencing depth. *In silico* simulations were performed to estimate model accuracy for a) α allele count and b) α duplication and c) $βIII^{FS}$ allele count models. Each color represents a dataset and for each dataset, model accuracy is estimated over 10 simulated samples at each sequencing depth. The average model accuracy -/+ standard deviation is shown.

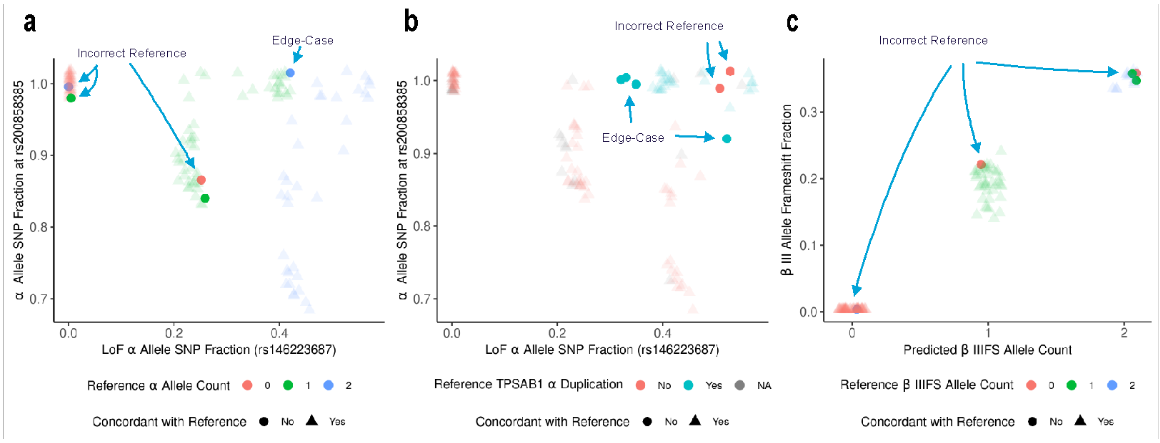

S6 Fig. Fractional abundance of selected *TPSAB1* and *TPSB2* polymorphisms compared with a) α allele count and b) α duplication and c) $βIII^{FS}$ allele count. Transparent triangles indicate concordance between the PCR NGS workflow and WGS or Sanger sequencing. Solid circles indicate samples where PCR NGS and WGS sequencing report different allele counts. Datapoints have been slightly jittered for clearer visualization. Some cases of discordance between the PCR NGS output and reference call can likely be attributed to inaccuracies in the reference method. These suspected cases are annotated as “incorrect reference”. A few examples of discordance between the two methods are edge-cases and more difficult to conclude.

S1a Table. Amplicon sequencing workflow.

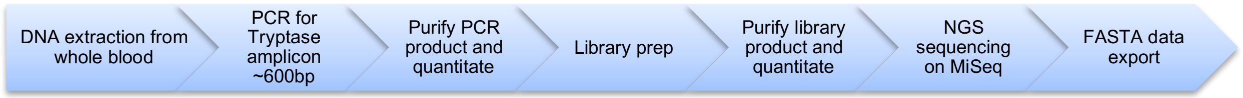

S1b Table. Primer design for α and β tryptase amplicon.

Forward primer (5’- 3’)	CTGTCCCCAGACCCACCATC
Reverse primer (5’- 3’)	CCCTCACCACATTCCACAGG

S1c Table. Primer design for α and β tryptase amplicon with adaptors.

Forward primer (5’- 3’)	5' TCGTCGGCAGCGTCAGATGTGTATAAGAGACAGCTGTCCCCAGACCCACCATC 3'
Reverse primer (5’- 3’)	5' GTCTCGTGGGCTCGGAGATGTGTATAAGAGACAGCCCTCACCACATTCCACAGG 3'

S1d Table. PCR conditions for primers and adaptors.

Temperature	Duration	Cycles
**95°C**	3 min	1
**98°C**	20 sec	35
**68°C**	15 sec	
**72°C**	30 sec	
**72°C**	5 min	1

S1e Table. Index library PCR conditions.

Temperature	Duration	Cycles
**72°C**	3 min	1
**95°C**	30 sec	1
**95°C**	10 sec	12
**55°C**	30 sec	
**72°C**	30 sec	
**72°C**	5 min	1

S2 Table. Tryptase genotype examples

Tryptase Locus on Chromosome 16p13.3	α Allele Count	α Duplication	βIII^{FS} Allele Count	Active β Allele Count (inferred)
βII — α<br>βII — βI<br>TPSB2 TPSAB1	1	No	0	3
βII — α<br>βIII^{FS} — α<br>TPSB2 TPSAB1	2	No	1	1
βII — α<br>βII — αα<br>TPSB2 TPSAB1	2	Yes	0	2
βIII^{FS} — αα<br>βIII^{FS} — βI<br>TPSB2 TPSAB1	1	Yes	2	1
βII — βI<br>βIII — βI<br>TPSB2 TPSAB1	0	No	0	4

S3 Table. Derivation of total number of α and β alleles from the PCR NGS output

	If α duplication is not present	If α duplication is present
Total α allele count	α	$\alpha + 1$
Total β allele count	4 - α	4 - α

α is the number of alleles reported by PCR NGS.

S4 Table. Accuracy of the PCR NGS tryptase genotyping workflow compared to ddPCR.

N = 51	total α allele count (ddPCR)				
derived total α allele count (prediction)		**0**	**1**	**2**	**3**
	**0**	15	0	0	0
	**1**	0	10	0	0
	**2**	0	3	17	1
	**3**	0	0	0	5

N = 51	βIII^{FS} allele count (ddPCR)			
βIII^{FS} allele count (prediction)		**0**	**1**	**2**
	**0**	32	0	0
	**1**	0	12	0
	**2**	0	0	7

N = 51	total β allele count (ddPCR)			
derived total β allele count (prediction)		**2**	**3**	**4**
	**2**	14	1	0
	**3**	2	19	0
	**4**	0	0	15
